# The Serial Mediating Role of Perceived Digital Cognitive Manipulation and Brain Rot in the Relationship Between Social Media Addiction and Resilience: An SEM and Network Analysis Study

**DOI:** 10.1002/brb3.71472

**Published:** 2026-05-05

**Authors:** Hasan Batmaz

**Affiliations:** ^1^ Department of Psychology Karabuk University Karabük Türkiye

**Keywords:** brain rot, network analysis, perceived digital cognitive manipulation, resilience, social media addiction, structural equation model

## Abstract

**Purpose:**

Social media continues to be associated with emerging adults’ thinking skills and decision‐making processes, as well as behavioral addictions. This research aims to examine the correlation between emerging adults’ levels of social media addiction and psychological resilience through the processes of “Perceived Cognitive Manipulation” and “Brain Rot,” and to develop an original Perceived Digital Cognitive Manipulation Scale (PEDCOM‐8) within this scope.

**Method:**

The research was conducted in two stages. In the first stage, confirmatory factor analysis was applied to data obtained from 151 participants in the pilot study and 309 participants in the confirmatory factor analysis conducted during the development of the PEDCOM‐8.

**Finding:**

The scale was found to be valid and reliable with a two‐factor structure and high internal consistency coefficients. In the second stage (N 355, mean age 20.78, 49.9% female), the relationships among social media addiction (X), perceived digital cognitive manipulation (M_1_), brain rot (M_2_), and resilience (Y) were examined using structural equation modeling (SEM) and network analyses, which were created with data obtained from 355 participants. The findings showed that social media addiction is a significant and strong predictor of perceived digital cognitive manipulation. It has been demonstrated that perceived digital cognitive manipulation is negatively associated with brain rot, and compromised brain health is, in turn, linked to lower levels of resilience. Serial mediation analysis results revealed that perceived digital cognitive manipulation and brain rot together play a significant mediating role in the relationship between social media addiction and resilience.

**Conclusion:**

The study presents a new conceptual model that explains the indirect roles of social media addiction on the cognitive integrity and psychological resilience mechanisms of emerging adults. The developed PEDCOM‐8 serves as a unique measurement tool for future research examining the mental effects of digital behaviors.

## Graphical abstract

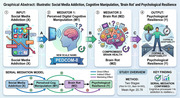

## 1 Introduction

Living in the digital age has become increasingly difficult. Perhaps individuals have adapted to this age too quickly. The psychological effects of this age, also known as the age of speed, have also begun to increase. While increasing social media use facilitates socialization, it also hurts mental health (Ergün et al. 2025; Feng et al. 2025; Nguyen et al. 2025). Excessive time spent on social media negatively impacts individuals' cognitive processes. The fact that individuals' avoidance‐ or reward‐oriented expectations, arising from rejection or popularity‐seeking behaviors on social media, can develop into addictive behaviors (Kaya et al. 2025; Zhao et al. 2024) underscores the severity of the risk. This finding suggests that spending extensive time on social media also weakens individuals' healthy, critical, and analytical thinking skills. In addition to the significant association between intense social media use and cognitive wear, it is also negatively linked to emotional processes. Fuentes Chavez et al. (2025) found a significant, positive correlation between social media addiction (SMA) and emotional regulation. In other words, as SMA increases, individuals' ability to regulate their emotions healthily weakens, making it particularly difficult to manage negative emotions and control impulses. J. Wang and Wang (2025)’s study revealed that SMA develops through the mechanism of emotional reinforcement. While positive reinforcement (likes, approval, a sense of reward) initially initiates addiction, over time, the introduction of negative reinforcement (escape from negative emotions, stress reduction) emotionally wears emerging adults down.

SMA, a type of behavioral addiction derived from Young's concept of “internet addiction” and defined by Andreassen specifically for social media, impairs a person's emotional regulation and cognitive control processes, impairing their social, academic, and professional functioning (Andreassen 2015; Andreassen et al. 2012, 2017; Young 1996, 1998). The impact of this addiction, not only on behavioral but also on cognitive thinking skills, is a recent topic of discussion. In particular, the Oxford Dictionary's declaration of “brain rot” as the word of the year for 2024 has forced researchers to focus on the cognitive processes affected by behavioral addiction. Brain rot (BR) has been described not as a clinical condition, but as a metaphorical form of cognitive exhaustion. Krebs (2025) found that SMA is associated with both a lack of cognitive control and the social gratification created by digital feedback (likes, emojis, etc.). It was determined that individuals with weak executive functions spend more time on social media, and these two factors together increase the risk of addiction. The findings of a different study revealed that high social media use and FOMO (fear of missing out) weaken attentional control and information evaluation processes by placing individuals under more intense cognitive load; this, combined with low cognitive control and high impulsivity, leads individuals to fail to critically evaluate the flow of information on social media and exhibit reactive behaviors (e.g., blocking or avoidance) (Ahmed et al. 2024).

As a result of intensive social media use, the concepts of “digital brain fatigue,” “information overload,” and “cognitive erosion” affect the “brain rot” process. Aka (2024) stated that intensive social media use increases cognitive load and emotional exhaustion over time, leading to “social network fatigue”. Constant interaction, distraction, and information overload at the addiction level lead to a loss of control and apathy in the user, thus creating a process that extends from addiction to fatigue. AI Heneidi and Smith (2021) demonstrated that excessive online information flow and social media use increase cognitive load, resulting in distraction, stress, and poor academic performance. Furthermore, a significant negative correlation was found between social network addiction and low well‐being. Sireli et al. (2023) demonstrated that problematic social media use (PSMU) is associated with increased cognitive distortions, which, in turn, lower self‐esteem. As shown, continuous and intensive social media use can reduce individuals’ cognitive capacity and negatively correlate with their cognitive health. Therefore, individuals’ mental health weakens, and their psychological resilience (RE) decreases (Doğrusever et al. 2026; Kulshreshtha 2025; R. Wang et al. 2025; Yam et al. 2024).

### 1.1 SMA and Perceived Digital Cognitive Manipulation: A New Conceptualization

SMA can be defined as a type of behavioral addiction characterized by an individual's uncontrolled, excessive, and functionally impairing use of social media platforms. This concept, previously coined as internet addiction by Kimberly Young (1996, 1998), later gained its theoretical basis in the “six‐component addiction model” (Salience, Mood, Modification, Tolerance, Withdrawal, Conflict, Relapse) developed by Mark D. Griffiths (2005). Andreassen adapted this model to the social media context and applied it in his studies, the Bergen Facebook Addiction Scale (BFAS) and the Bergen Social Media Addiction Scale (BSMAS) (Andreassen et al. 2012). Numerous studies have been conducted in the past regarding the impact of SMA, a behavioral addiction, on psychological mechanisms.

The effects of SMA on individuals are showing new trends evolving from behavioral orientations to cognitive processes. One of the trends addressed in this study is “perceived digital cognitive manipulation,” which refers to changes in an individual's thought patterns resulting from repetitive, manipulative, and guiding content on social media. Individuals are constantly exposed to emotionally charged, repetitive, and algorithmically guided content on social media. Over time, this intense exposure can affect not only superficial information flow but also the individual's fundamental cognitive patterns—how they make sense of the world, themselves, and others. In this context, rather than referring to a coercive or clinical process, perceived digital cognitive manipulation (PEDCOM) reflects the implicit reshaping of an individual's thought patterns through continuous exposure to manipulative digital content. As individuals are repeatedly exposed to algorithm‐driven stimuli, they may become more susceptible to external influence and online conformity. Consequently, this continuous exposure can gradually weaken critical evaluation skills, leading to a subtle realignment of personal beliefs and attitudes with external digital messages. This concept represents a different plane from the direct, coercive ideological indoctrination processes found in political psychology and sociopolitical literature. The “perceived digital cognitive manipulation” conceptualized in this study does not reflect a macro‐level political imposition; rather, it reflects the cognitive sensitivity and vulnerability that individuals feel towards persuasive, guiding, and manipulative content in the digital world. Since no existing psychometric tool can evaluate this specific structure, newly introduced in the context of social media, and individuals’ self‐perceptions regarding it, the need arose to develop a new measurement tool.

### 1.2 Brain Rot

One concept closely associated with PEDCOM in this research model is BR. The widespread use of the internet and social media has led to radical changes in individuals' cognitive functions. This environment has a structure that raises the threshold of mental arousal, shortens attention span, and reinforces a state of constant alertness (Loh and Kanai 2016). This transformation is paving the way for the emergence of new phenomena that threaten psychological well‐being. In this context, the concept of BR, chosen as the “word” of 2024 by Oxford University (Oxford University Press 2024), stands out as a striking definition that characterizes the potential negative correlation of digital content consumption with individual psychological health. The concept of “brain rot” in this context is not a clinical picture; rather, it is a metaphorical construct that conveys mental fatigue and cognitive exhaustion resulting from excessive and manipulative exposure to digital stimuli. Some scales have been developed to measure BR in Turkish samples (Batmaz et al. 2025; Yılmaz and Aktürk 2025). Yousef et al. (2025) emphasized that when these elements combine, social media and short digital content cause excessive executive outputs such as attention, memory, and decision‐making, and this process accelerates compulsive processes such as “doomscrolling” and “zombie scrolling,” highlighting that “brain rot” is a significant health warning sign requiring clinical diagnosis and treatment. The process by which uncontrolled social media use impairs individuals' attention, memory, and decision‐making skills, and negatively correlates with the output of critical and analytical thinking, has been termed “brain rot.” BR has been shown to pose a threat to individual RE and a risk to social cohesion and productivity from a social perspective (Al Husaini 2025).

### 1.3 Resilience

Many researchers have defined the concept of RE. It is a dynamic, contextual, and developmental process that enables an individual to adapt positively despite significant stress or adversity; it is based on “the normal growth and regulatory capacities of human nature” (Masten and Reed 2002, 75–76). They define it as “a dynamic process based on the interaction of the individual and environmental systems through which positive adaptation occurs despite adversity” (Riley and Masten 2005, 14). Alternatively, they define it as “the capacity of an individual to withstand, adapt, and recover from exposure to stress, trauma, disaster, or significant risk factors” (Connor and Davidson 2003, 76). RE is understood to act as a protective shield for mental health. However, as a result of intensive and uncontrolled use of social media, individuals' RE levels weaken (Almulla et al. 2025; Ataman‐Bor et al. 2025; Bağatarhan 2025). High digital stress has also been shown to negatively impact RE (Qi and Yang 2024). Previous research confirms the negative impact of SMA on RE (Çiçek et al. 2025; Lin et al. 2024; Nabil Abu El‐Fadl et al. 2024; Kocabıyık and Bacıoğlu 2022; Sarwar et al. 2025).

### 1.4 Present Study

There are no studies in the literature that quantitatively test the association between “perceived digital cognitive manipulation” and BR (cognitive exhaustion). Studies on SMA have focused on behavioral aspects, but the mechanisms of cognitive dimensions have not been adequately examined. RE has not been considered as an outcome variable. Therefore, this study is the first to test the relationship between SMA → PEDCOM → BR (cognitive exhaustion) → RE using a serial mediation model.

The primary objective of this research is to develop a valid and reliable psychometric measure of the phenomenon of “perceived digital cognitive manipulation” on social media (Study 1). This potential aims to expand the scope of this scale, enabling the assessment of persistent and manipulative content across digital boundaries in a more objective and extensible manner. In Study 2, this scale will be used to examine the distributions among the variables of SMA, PEDCOM, BR, and RE.

### 1.5 Research Hypotheses

H1: SMA positively predicts PEDCOM and BR.
H2: Brainwashing (BW) positively predicts BR.
H3: PEDCOM and BR negatively predict RE.
H4: PEDCOM mediates the relationship between SMA and RE.
H5: PEDCOM and BR sequentially mediate the relationship between SMA and RE.

## 2 Method

In this study, data were collected using convenience sampling. While practical, convenience sampling is susceptible to sampling bias and systematic errors (Alvi 2016). The study sample consisted of emerging adults with diverse demographic characteristics, which raises the possibility that subgroups may not fully represent one another. So, the research findings should be considered limited to this sample and not generalized to the general population.

This study was conducted in two parts: Study 1 and Study 2. Study 1 included analyses of the production of perceived digital cognitive manipulation (PEDCOM‐8) in Turkish (validity and reliability). Study 2 examined the structural distortions (SEM) between the concepts of SMA, psychological RE, BR, and BW (Kline 2023). Analyses of the PEDCOM‐8 and SEM models were conducted using data from different parts.

## 3 Study 1

### 3.1 Development Process of PEDCOM‐8

PEDCOM, as a psychological term, refers to the process of changing individuals’ thoughts, beliefs, or behaviors in a coercive or manipulative way. Within the scope of Study 1, the validity and reliability of the PEDCOM‐8 were analyzed using Turkish data. Study 1 employed a quantitative research design, which enabled numerical analysis of the data. Quantitative designs are widely preferred, particularly in scale development, to obtain reliable findings (Creswell 2014). A cross‐sectional survey design was chosen in accordance with the research purpose. First, to develop the PEDCOM‐8 classes, the literature was reviewed and emerging issues identified. This skill has a 22‐item pool. These items were reviewed based on the opinions of four experts with doctoral degrees in psychology, as well as guidance and psychological counseling. Similar items were combined to reduce the total to 12 items. A pilot study was then conducted using the 12‐item scale (*n* = 151; 57% female; mean = 20.55, SD = 4.75). Following validity analyses of the pilot studies, two items with values below 0.40 were removed from the dimension.

**FIGURE 1:**
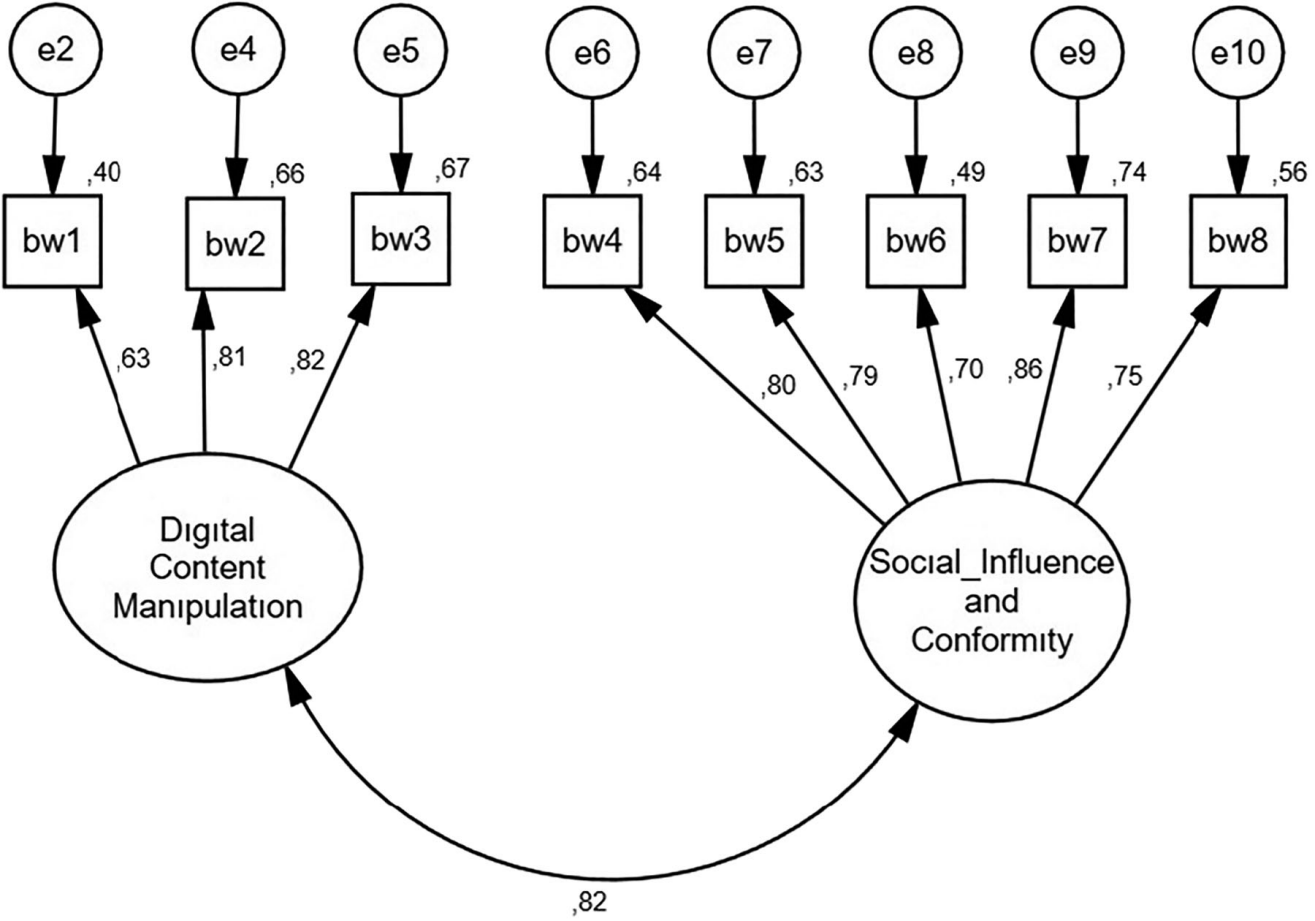
CFA of the PEDCOM‐8.

Following the pilot study, data were collected for a confirmatory factor analysis (CFA) on the 10 identified items (*N* = 309). As a result of the CFA, two items that compromised the model fit and had loadings below 0.40 were removed, and the scale was finalized with eight items, consisting of two dimensions: Digital Content Manipulation (items 1–3) and Social Influence and Conformity (items 4–8) (see Figure 1). The model fit values obtained after the CFA are as follows: CMIN/df 1.914, *p* < 0.05, CFI 0.987, TLI 0.981, IFI 0.987, RMSEA 0.054, SRMR 0.028. The construct demonstrated adequate reliability and convergent validity, with a composite reliability (CR) of 0.90 and an average variance extracted (AVE) of 0.55, both exceeding the recommended thresholds of 0.70 and 0.50, respectively. The Fornell–Larcker criterion was applied to test the discriminant validity between the sub‐dimensions. The analysis revealed that the AVE value of digital content manipulation dimension (0.79) was higher than the inter‐dimensional correlation (0.70). Although the AVE value of social influence and conformity dimension (0.69) was slightly below the correlation coefficient (0.70), considering the high CR values of both dimensions (0.83 and 0.77) and the theoretically mutually reinforcing nature of the structures, the inter‐dimensional discriminant validity was deemed to be at an acceptable level. Finally, the PEDCOM‐8 was converted into an 8‐item scale with a maximum score of 40 and a minimum score of 8.

Data for the scale, comprising 355 participants (49.9% female), were collected through exploratory factor analysis (EFA) and the SEM model. Analyses of these data revealed that Cronbach's alpha (*α*) was 0.85 and McDonald's Omega (*ω*) was 0.85.

According to Table 1, factor loadings (structure matrix) were determined to be 0.78, 0.83, 0.75, 0.82, 0.51, 0.47, 0.87, and 0.79, respectively (Extraction = Principal components, Rotation = Promax). Principal component analysis (PCA) was used to size this high‐performance novel structure. While classical common factor analysis aims to reveal latent traits among its elements, PCA summarizes the data by listing fewer elements while maximizing the explanation of the total variance (including common, specific, and error variance) (Costello and Osborne 2005; Pett et al. 2003). This effective “perceived systematic regime” was first brought together to perform empirical data reduction while preserving the total variance of systematic objective groups and to identify the items that form the strongest whole at the compositional level. Information regarding the specification of the scale's criteria was provided in the introduction of the SEM model in Study 2. Based on these results, the PEDCOM‐8 was found to be a valid and reliable scale in Turkish samples.

**TABLE 1:** PEDCOM‐8 and descriptive statistics.

Scale items	Factor loads	Mean	SD
1. The people or groups I follow on social media change my thoughts and behaviors	0.78	2.51	1.31
2. Content on social media makes me doubt my thoughts	0.83	2.42	1.32
3. Social media posts can negatively impact my decision‐making	0.75	2.01	1.16
4. Instead of expressing my opinions, I conform to the views of those around me	0.82	1.80	1.16
5. People constantly imposing the same ideas affects my thoughts/behaviors	0.51	2.15	1.27
6. The people around me are indirectly dictating what I should think	0.47	2.32	1.26
7. Sometimes, the opinions of my social circle and friends can be more influential than my own thoughts	0.87	1.86	1.15
8. I have difficulty distinguishing between my own thoughts and those of others	0.79	1.87	1.18

*Note*: Total variance = %48.55.

## 4 Study 2

Part 2 of the study examined the serial roles of the concepts of PEDCOM and BR, which mediate the impact of SMA on psychological RE. First, descriptive statistics were used to identify the relationships between the concepts. The model was then tested.

### 4.1 Participants

This study included 355 participants. The mean age of the participants was 20.78 (SD 2.49), with ages ranging from 18 to 24. The study comprised 49.9% female and 50.1% male participants. Around 83.1% of the participants had an undergraduate degree, 11.5% had a high school degree, and 5.4% had a postgraduate degree. The daily social media usage duration of the participants was 1–3 h (28.5%), 3–5 h (39.7%), 5–7 h (20.8%), and 7–9 h and above (11%). In addition, the participants' social media platforms were Instagram (73.8%), TikTok (16.1%), X (8.5%), and Facebook (1.7%). All participants volunteered for this study.

### 4.2 Measures

#### 4.2.1 Perceived Digital Cognitive Manipulation Scale (PEDCOM‐8)

Developed to determine the extent to which external influences shape individuals' thoughts, attitudes, and behaviors, the PEDCOM‐8 measures an individual's openness to manipulation across cognitive, emotional, and behavioral levels. The scale consists of 8 items across two subscales: Digital Content Manipulation (items 1–3) and Social Influence and Conformity (items 4–8). Participants respond to each item on a 5‐point Likert‐type scale (1 = *Strongly disagree*, 5 = *Strongly agree*). Higher scores on the scale indicate a greater tendency for individuals to be influenced by external factors, both cognitively and emotionally. In this study, the internal consistency coefficient of the scale was found to be (Cronbach's *α* = 0.85).

#### 4.2.2 Brain Rot Scale (BRS‐8)

Developed by Batmaz et al. (2025) to assess cognitive and psychological burnout, the BRS‐8 consists of 8 items and two subscales (cognitive fatigue and mental burnout). EFA revealed that the factor loadings of the items ranged from 0.53 to 0.80, with an explained variance (AVE) of 0.53 and a CR of 0.90. The scale has a high level of internal consistency (*α* = 0.83; *ω* = 0.83). Criterion validity analyses found that the BRS‐8 showed positive and significant relationships with SMA (*r* = 0.50, *p* < 0.05) and FOMO (*r* = 0.28, *p* < 0.05). CFA indicated a good model fit (CMIN/df = 2.34; RMSEA = 0.043; CFI = 0.986; TLI = 0.978; SRMR = 0.027). These findings demonstrate that the BRS‐8 is a psychometrically valid and reliable instrument. In the current study, Cronbach's alpha coefficient for the scale was calculated as *α* = 0.85.

#### 4.2.3 Brief RE Scale

The scale was developed to measure an individual's ability to “recover” from stressful situations (Smith et al. 2008). The scale consists of six items (e.g., “It doesn't take me long to recover from stressful situations”), and each item is rated on a 5‐point Likert scale ranging from 1 (strongly disagree) to 5 (strongly agree). A higher score on the scale indicates a higher level of RE. The scale was translated into Turkish by Doğan (2015). In this study, Cronbach's *α* was 0.74.

#### 4.2.4 Bergen Social Media Addiction Scale

Developed by Andreassen et al. (2017), the BSMAS was created to measure addiction levels related to social media use. The scale consists of six items, and participants respond to each item on a 5‐point Likert‐type scale, ranging from 1 (very rarely) to 5 (very often). Items include statements such as “Did you have difficulty quitting social media?” or “Did you experience problems due to your social media use?” The original form of the scale exhibits high internal consistency (Cronbach's *α* = 0.88). Its Turkish adaptation, conducted by Demirci (2019), yielded a Cronbach's *α* value of 0.82. The scale contains no reverse items, indicating that higher scores correspond to higher levels of SMA. In this study, Cronbach's *α* for the scale was 0.81.

### 4.3 Data Analysis

The obtained data were analyzed using SPSS 27.0, AMOS 24.0, and RStudio (version 4.5.2) programs. Scale reliability was assessed using Cronbach's alpha coefficients, and structural stability was evaluated using CFA. The model's fit was evaluated using CMIN/df, CFI, TLI, IFI, and RMSEA. The study investigated the robustness of social media sex, its direct and indirect roles on PEDCOM, and BR using SEM.

## 5 Results

Before proceeding to SEM analysis, correlation analysis was conducted to examine relationships among variables, and *t*‐tests and ANOVA were performed to investigate differences across demographic variables. All presented coefficients are standardized. SMA was positively correlated with PEDCOM and BR. SMA was significantly negatively correlated with psychological RE. Furthermore, PEDCOM was positively correlated with BR and SMA and significantly negatively correlated with psychological RE (see Table 2).

**TABLE 2:** *t*‐test results of variables by gender and correlation analysis.

Variable	Gender	*n*	*X̄*	SD	*t*	df	*p*	1	2	3	4
1. SM	Female	177	17.86	5.71	3.079*	353	0.001				
	Male	178	15.96	5.89				—			
2. PEDCOM	Female	177	17.62	6.72	1.781	353	0.076	0.39*	—		
	Male	178	16.33	6.92						—	
3. BR	Female	177	24.37	7.49	3.624*	353	0.001	0.63*	0.49*		
	Male	178	21.34	8.24							
4. RE	Female	177	17.96	4.63	−4.466*	353	0.001	−0.26*	−0.34*	−0.36*	—
	Male	178	20.30	5.20							

*p < 0.05, *N* = 355.

According to the *t*‐test results in Table 2, SMA levels are significantly higher in women (*X̄* = 17.86) than in men (*X̄* = 15.96) (*t* = 3.079, *p* < 0.01). PEDCOM scores appear to be higher in women than in men; however, the difference is not statistically significant (*p* = 0.076). BR levels are significantly higher in women (*X̄* = 24.37) than in men (*X̄* = 21.34) (*t* = 3.624, *p* < 0.01). RE scores are significantly higher in men (*X̄* = 20.30) than in women (*X̄* = 17.96) (*t* = −4.466, *p* < 0.01). Consequently, female participants score higher in SMA and BR, while male participants show higher levels of psychological RE. No significant difference was found between genders in the PEDCOM variable.

The ANOVA in Table 3 reveals significant differences across all variables based on duration of daily social media use (*p* < 0.05). SMA scores significantly increase with duration of use (*F* = 20.83, *p* = 0.000). PEDCOM levels vary with the duration of use (*F* = 4.59, *p* = 0.004). The biggest difference was observed in the BR variable, with significantly higher scores for long‐term users (*F* = 7.87, *p* < 0.001). RE varies in the opposite direction with duration of use, with RE levels significantly decreasing for longer‐term social media users (*F* = 3.37, *p* = 0.019). Consequently, the relationship between duration of social media use, PEDCOM, and BR increases, while RE decreases.

**TABLE 3:** ANOVA results based on daily social media usage duration.

Variable	Variance source	Sum of squares	SD	Mean square	*F*	*p*
SM	Between groups	148.72	3	616.24		
	Within groups	10379.57	351	29.57	20.83*	0.000
	Total	12228.29	354			
PEDCOM	Between groups	627.22	3	209.076		
	Within groups	15963.54	351	45.48	4.59*	0.004
	Total	16590.77	354			
BR	Between groups	1433.01	3	477.67		
	Within groups	21281.36	351	60.63	7.87*	0.000
	Total	22714.38	354			
RE	Between groups	253.73	3	84.57		
	Within groups	8810.50	351	25.10	3.37*	0.019
	Total	9064.32	354			

*p < 0.05.

### 5.1 Direct Roles of SMA on RE

**FIGURE 2:**
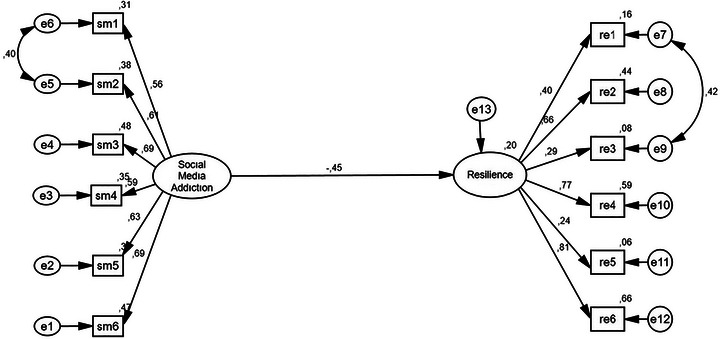
SEM diagram for first model.

The first model was created to examine the role of SMA (independent variable) on psychological RE (dependent variable). The model diagram is shown in Figure 2.

**FIGURE 3:**
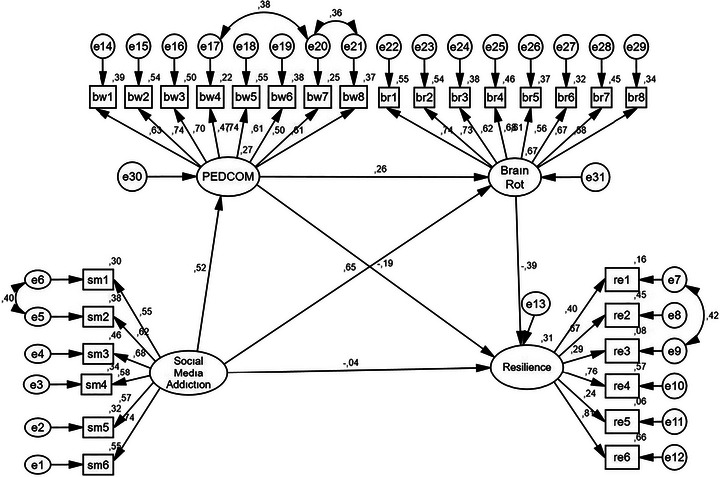
SEM diagram including mediating variables.

The first model fit indices are as follows: *χ* ^2^/df = 2.856, RMSEA = 0.072, CFI = 0.92, GFI = 0.94, and SRMR = 0.068. In the analysis, covariances were created for the first model fit indices through the binary residual terms (e5–e6 and e7–e9). The SMA variable explained 20 % of the variance in RE. The path diagram including the mediating variables is shown in Figure 3.

The regression weights shown in the diagram obtained by including the mediator variables in the model were statistically significant (*p* < 0.001). The structural model, including the mediator variables (see Figure 3), revealed that SMA significantly predicted PEDCOM (*β* = 0.52; *p* < 0.001). The model fit values obtained as a result of the path analysis, including the mediator variables in the model, are as follows: *χ* ^2^ /df 2.086, RMSEA 0.055, CFI 0.90, GFI 0.87, and SRMR 0.057 (Figure 3). The goodness of fit values are seen to be sufficient. A bootstrap analysis was used to examine whether PEDCOM and BR mediate the relationship between SMA and RE. The results of the bootstrap analysis are shown in Table 4.

**TABLE 4:** The results of bootstrapping analyses.

Model pathways	Coefficient	Lower	Upper
**Standardized direct roles**			
Social media addiction → PEDCOM	0.52	0.40*	0.63*
Social media addiction → Brain rot	0.65	0.52*	0.78*
PEDCOM → Brain rot	0.26	0.12*	0.40*
PEDCOM → Resilience	−0.19	−0.35*	−0.03*
Brain rot → Resilience	−0.39	−0.67*	−0.13*
Social media addiction → Resilience	−0.04	−0.30**	0.24**
**Standardized indirect roles**			
Social media addiction → Resilience	−0.25	−0.64*	−0.22*

*p < 0.01, **p > 0.05.

An examination of Table 4 reveals that the bootstrap analysis indicates that the path coefficients (direct roles) are statistically significant (*p* < 0.01). Furthermore, the indirect roles were also determined to be significant (bootstrap coefficient = −0.25, 95% CI = [−0.64, −0.22], *p* < 0.01). These findings reveal that the indirect role of SMA on psychological RE through PEDCOM and BR is significant. According to the results, PEDCOM and BR variables fully mediate the relationship between SMA and psychological RE (standardized direct effect = −0.04, 95% CI = [−0.30, 0.24], *p* > 0.05). This results in the full mediating role of these two variables.

### 5.2 Psychological Network Structure and Centrality Findings

In the network analysis, the centrality values of the variables included in the model (SM, PEDCOM, BR, RE) were examined. The fact that all betweenness coefficients were zero indicates that the network operates in a linear chain. This is consistent with the structure of the serial mediation model (SMA → PEDCOM → BR → RE) hypothesized in the study. According to the network centrality analysis, BR has the highest closeness and strength among the variables (closeness = 0.468; strength = 1.48). This finding indicates that BR is the node that most strongly interacts with other variables in the network. While the variables SMA and PEDCOM exhibit moderate centrality, durable shows low values for both closeness (0.314) and strength (0.96). If the value between all variables is zero, the role of network policy is evident.

The strength values of the SMA and PEDCOM variables were moderate (1.28 and 1.22, respectively), indicating that these variables serve as entry and interconnection points in the model. While the SM variable represents the starting point of the process, the PEDCOM variable serves as an intermediate link in transferring the influence from the SM to the BR. Both the closeness (0.314) and strength (0.96) values of the RE variable were lower than those of the others. This result indicates that RE is the terminal node of the network, the outcome variable reached by the effects in the system.

**FIGURE 4:**
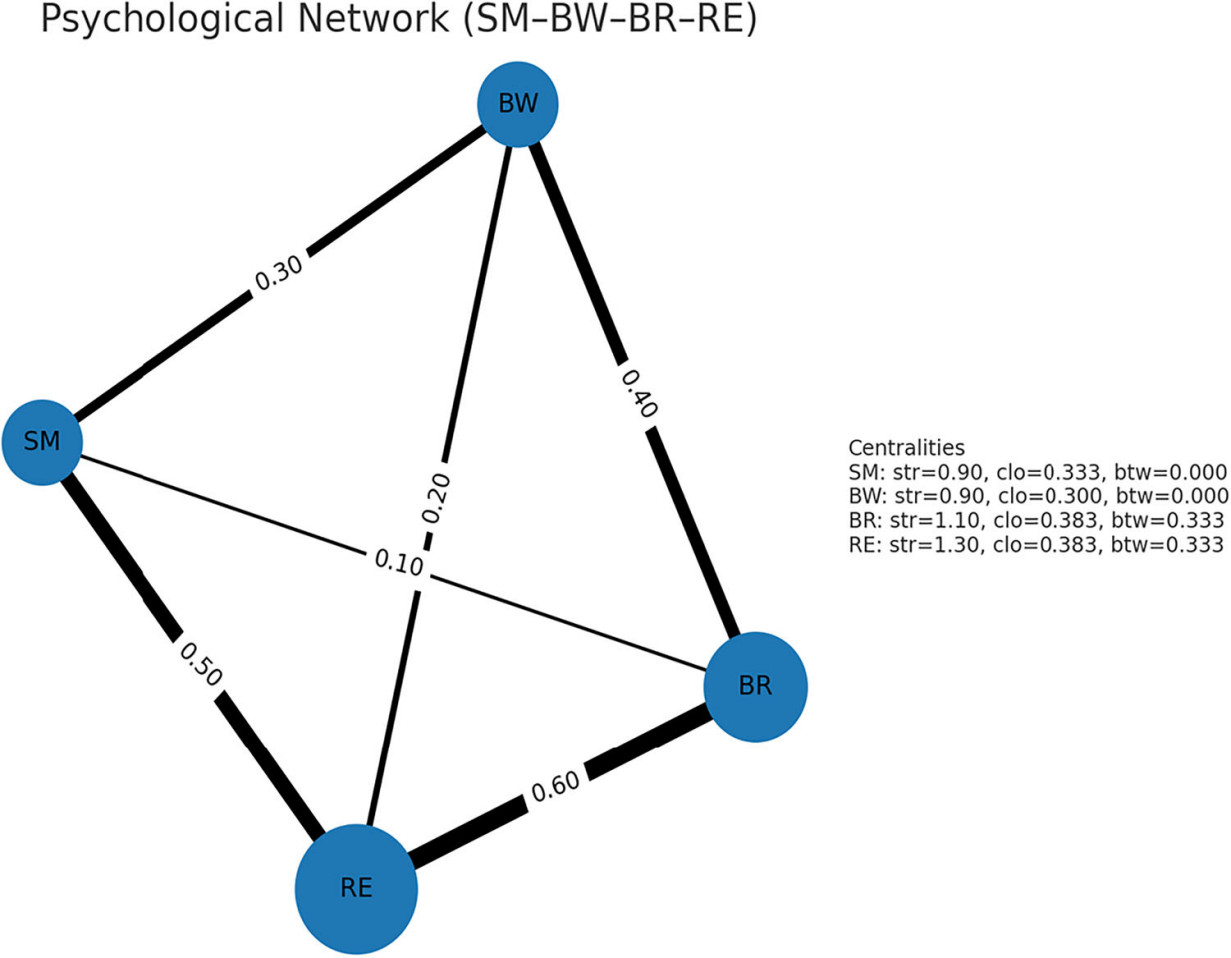
Weighted network structures showing the relationships between variables. (Note: BW = PEDCOM, edge thickness = weight [0.10–0.60], node size = node strength [total bond strength]. You can see the strength, closeness, and betweenness values for each node in the box on the right).

Overall, the network structure reveals that SMA is indirectly associated with RE through the interconnecting roles of PEDCOM and BR. This points to a network topology that supports the study's serial mediation hypothesis (see Figure 4).

## 6 Discussion

The results confirmed the hypotheses developed to explore relationships between SMA and cognitive and psychological processes, revealing mediating roles among the variables. After testing the hypotheses discussed in the study, SMA emerged as a central correlating variable with a significant, negative association with RE. Furthermore, the indirect pathways involving PEDCOM and BR, which bridge the relationship between SM and RE, were identified.

The first finding of the research is that SMA is negatively associated with RE. Previous studies confirm this relationship (Çiçek et al. 2025; Lin et al. 2024; Nabil Abu El‐Fadl et al. 2024; Kocabıyık and Bacıoğlu 2022; Sarwar et al. 2025). It is understood that emerging adults who spend excessive time on social media experience deterioration in their mental health and, consequently, weaken RE, which acts as a “shield.” In a study on this subject, it was found that SMA reduces perceived social support, and this decreased social support negatively correlates with RE (Bilgin and Taş 2018). As emphasized by González‐Nuevo et al. (2022), social media use is significantly and negatively associated with individuals' cognitive processes and psychological RE. In line with the results obtained by Hidalgo‐Fuentes et al. (2023), it is revealed that SMA and problematic internet use are negatively associated with individuals' RE. These results suggest that SMA shares a negative link with psychological RE, through a different psychological mechanism. In other words, emerging adults and university students who exhibit SMA or extensive use may experience increased loneliness, weakened social support networks, and decreased levels of psychological well‐being, which in turn shares a negative link with their psychological RE.

Another finding of the study revealed the serial mediating roles of PEDCOM and BR in the relationship between SMA and RE. Because PEDCOM and BR are new concepts, no studies have been found linking them. The process of PEDCOM in this study aligns with the literature on how PSMU, echo chambers, and exposure to conspiracy theories can heighten an emerging adult's digital susceptibility; the current findings are consistent with this line of research. Individuals with PSMU are found to have higher levels of engagement and belief in fake news (Meshi and Molina 2025). This is one of the findings most closely aligned with the PEDCOM dimension, indicating that emerging adults’ weakened cognitive defenses are a key factor in this context. In a separate study, when cognitive reflection is low, the ability to distinguish misinformation on social media is impaired, thereby increasing susceptibility to persuasion and manipulation processes (Ali and Qazi 2022). Using Facebook/YouTube for news has been shown to increase conspiracy beliefs (even when controlling for cognitive reflection), which falls under the “belief reframing” aspect of PEDCOM. In the context of the pandemic, “identity‐based conspiracy bubbles” have been reported to have been reinforced on social media, leading to increasingly extreme beliefs (Stecula and Pickup 2021). These results support that SMA is negatively associated with psychological RE through PEDCOM → BR.

The network analysis results support the structural validity of the sequential mediation model proposed in the study. The betweenness values of all variables being zero indicate that the network operates in a linear chain, consistent with the serial mediation construct of SMA → PEDCOM → BR → RE. This finding suggests that the flow of information and influence within the network's central nodes is direct, while indirect or peripheral connections are weak (Epskamp et al. 2018). The closeness and strength values reveal that the variables play different roles in the network's interaction intensity. In particular, the BR variable has the highest closeness (0.468) and strength (1.48) values, indicating that this variable serves as a central node in the transfer of information and influence and, therefore, acts as an intermediary link connecting the cognitive and psychological consequences of SMA. Conversely, the lowest centrality values for the RE variable confirm its position as a peripheral node in the network, rather than a central hub, and indicate that it is strongly associated with the other variables. Therefore, network analysis findings indicate that the causal flow assumed in the structural equation model (Costantini et al. 2019) is empirically supported.

In the final analysis, *t*‐tests and ANOVA were conducted to determine whether the main variables differed by gender and daily social media use. This finding aligns with previous studies (Arı and Çarkıt 2020; Choudhury and Ali 2020; Gök and Yılmaz Koğar 2021; Horrich et al. 2024; Shannon et al. 2022; Selvaraj 2020). The ANOVA results reveal significant differences among all variables in daily social media usage duration (*p* < 0.05).

### 6.1 Limitations

This research has several limitations. Because the study was conducted only on university students within a specific age range, it is not possible to generalize the results to the general population or to different age and socioeconomic groups. First, because this study is cross‐sectional, it is not possible to establish temporal precedence or draw definitive causal conclusions among the variables. Furthermore, the fact that the majority of participants in the sample shared the same cultural background limits cross‐cultural comparisons of the findings. Because the study used self‐report measures, participants' subjective assessments, social desirability bias, and their tendency to respond honestly may have influenced the results. Assessing abstract and cognitive processes, such as “perceived digital cognitive manipulation” and “brain rot,” with these measurement tools raises concerns about conceptual accuracy. Another limitation of the study is its cross‐sectional design. Therefore, directional relationships between variables can only be interpreted in terms of structural fit, not in a causal context. Another limitation of this study concerns the sample characteristics: It consists entirely of university students in Türkiye, with a mean age of 20.78. Given that this group represents a unique developmental stage (emerging adulthood) and a specific cultural context, the findings are only directly relevant to this population. Therefore, these results should not be generalized to the broader public or different cultures, and future studies are encouraged to use more diverse and cross‐cultural samples to test the generalizability of these structural relationships.

## Author Contributions

**Hasan Batmaz**: conceptualization, methodology, writing – original draft, formal analysis.

## Funding

The study was supported by the Scientific and Technological Research Council of Türkiye.

## Ethics Statement

This study was conducted in accordance with the ethical standards of the Declaration of Helsinki. Ethical approval was obtained from the Ethics Committee of Karabuk University (approval no: 25, date: October 10, 2025).

## Consent

Written informed consent was obtained from all participants before their inclusion in the study.

## Conflicts of Interest

The author declares no conflicts of interest.

## Data Availability

The datasets used and/or analyzed during the current study are actually available from the corresponding author when you'd like.
